# Operando IR Optical Control of Localized Charge Carriers in BiVO_4_ Photoanodes

**DOI:** 10.1021/jacs.3c04287

**Published:** 2023-08-01

**Authors:** Zhu Meng, Ernest Pastor, Shababa Selim, Haoqing Ning, Marios Maimaris, Andreas Kafizas, James R. Durrant, Artem A. Bakulin

**Affiliations:** † Department of Chemistry and Centre for Processible Electronics, Imperial College London, London W12 0BZ, United Kingdom; ‡ IPR−Institut de Physique de Rennes, CNRS-Centre National de la Recherche Scientifique, UMR 6251 Université de Rennes, 35000 Rennes, France; § London Centre for Nanotechnology, Imperial College London, London SW7 2BP, United Kingdom

## Abstract

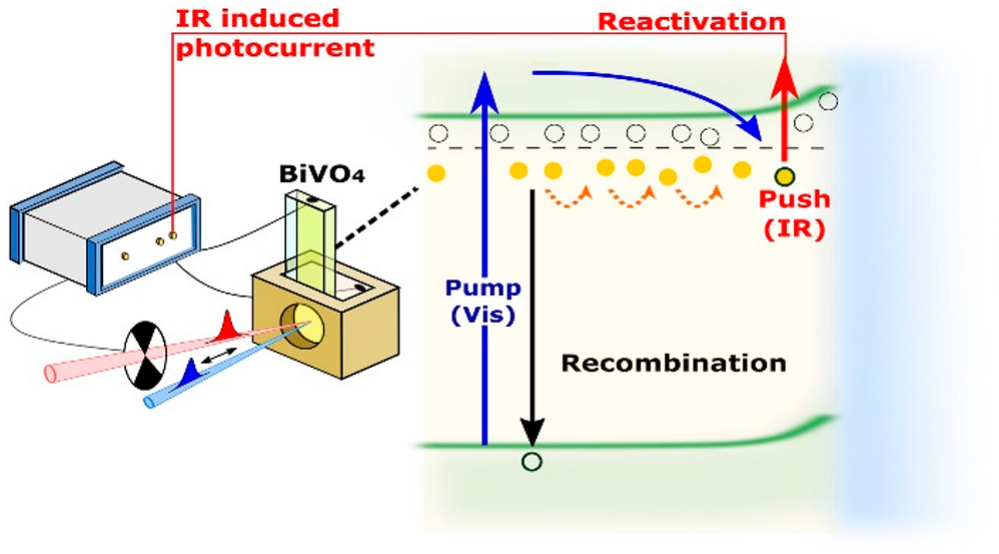

In photoelectrochemical cells (PECs) the photon-to-current conversion efficiency is often governed by carrier transport. Most metal oxides used in PECs exhibit thermally activated transport due to charge localization via the formation of polarons or the interaction with defects. This impacts catalysis by restricting the charge accumulation and extraction. To overcome this transport bottleneck nanostructuring, selective doping and photothermal treatments have been employed. Here we demonstrate an alternative approach capable of directly activating localized carriers in bismuth vanadate (BiVO_4_). We show that IR photons can optically excite localized charges, modulate their kinetics, and enhance the PEC current. Moreover, we track carriers bound to oxygen vacancies and expose their ∼10 ns charge localization, followed by ∼60 μs transport-assisted trapping. Critically, we demonstrate that localization is strongly dependent on the electric field within the device. While optical modulation has still a limited impact on overall PEC performance, we argue it offers a path to control devices on demand and uncover defect-related photophysics.

## Introduction

Optimal charge carrier transport pathways are crucial to improving photon-to-current conversion efficiencies in optoelectronic devices. While many of the semiconductor employed in solar cells sustain delocalized charges promoting fast band transport, most metal oxides used in photoelectrochemical cells (PECs) exhibit thermally activated transport.^1^ In these systems charges localize, electronically and spatially, generating new states within the band gap, away from the bands. Charge localization in photocatalytic oxides like Fe_2_O_3_, TiO_2_, or BiVO_4_ has been reported to happen via the formation of polarons,^2−6^ a structural distortion associated with a charge, or via the interaction with native defects inducing trap states or defect-bound polarons.^7,8^

These charge localization processes impact PEC performance by limiting the attainable Fermi level splitting,^9^ and consequently limit the reactions that can be photodriven, and by restricting charge movement inducing a transport bottleneck.^10^ Importantly, slow transport hinders charge separation and prevents the accumulation of charges at the electrochemical interface, potentially influencing electron transfer steps and even altering reaction mechanisms.^11^ Such broad impact highlights the importance of controlling charge transport pathways and has been the driver of intense research since the first reports of photoelectrochemical activity.

Among all native defects and dopants, oxygen vacancies in metal oxide photoelectrodes have been observed to play a critical role in transport and overall device efficiency.^12^ The absence of an oxygen atom in the oxide’s structure results in the formation of subvalent metallic sites, which can give rise to sub-band gap states responsible for n-type doping.^10^ Increasingly, research efforts have been devoted to exposing the underlying mechanisms behind the generation of mobile carriers and elucidating how vacancy control can affect and improve the process.

From an experimental viewpoint, the role of vacancies has been studied by electrochemical techniques such as impedance spectroscopy^13−20^ and computational studies which show electronic structure adjustments when vacancies form.^17,21,22^ In addition, optical spectroscopy measurements, including time-resolved photoluminescence and absorption spectroscopies, have suggested that vacancy-associated states enable charge hopping pathways and can act as centers for trap-assisted recombination, leading to reduced quantum yields.^23−25^

However, optical time-resolved measurements are not regularly performed under PEC operation conditions and, therefore, do not provide device-relevant information. Indeed, there are often questions as to whether the pulsed excitation conditions employed in these measurements generate realistic dynamical states that present under steady-state operation. This makes it difficult to ascertain whether the observed deactivation paths are actually relevant for the working devices. Moreover, during PEC working conditions, an inhomogeneous field distribution is present in the photoelectrode, affecting charge carrier generation and separation. Yet, traditionally, spectroscopic studies have struggled to reliably distinguish between mobile and trapped carriers as well as between carriers trapped at the bulk or in the reactive space charge layer. This has limited the impact that these measurements can have on guiding device improvements.

From a synthetic/engineering viewpoint, transport and defect control has been achieved with multiple strategies including altering the material’s nano- and crystal structure to minimize transport lengths,^26−30^ incorporating n-type dopants,^31−35^ or surface cocatalysts^30,36−39^ and even passivating defect states.^20,40−43^ For BiVO_4_, one of the best performing photoanodes where transport is a key limiting factor,^44^ Abdi et al. reported using a gradient doping method to maximize charge separation.^45^ Similarly, a recent study demonstrated how tailored control of phosphorus doping could be used to synthetically modulate carrier densities, change polaron transport, and ultimately improve extraction yields.^46^

It is generally established that careful tuning of the concentration of oxygen vacancies through chemical or thermal treatments can result in enhancements of PEC performace.^39,41^ In a recent study, it was shown that oxygen vacancies in BiVO_4_ extend over 600 meV below the conduction band and could be thermally activated to a mobile state by overcoming a 200 meV barrier. Similarly, it has been demonstrated that photothermal activation of vacancies though device heating could be used as a strategy to both employ the IR solar spectrum and enhance carrier collections.^47^ Despite these important advances, the underlying strategies rely on permanent changes to the sample or to the reaction conditions and, therefore, offer only a static control of the transport. This significantly limits the adaptability of the system to the changing electrochemical and illumination conditions that a device would experience. Recently, it was shown that IR photons could resonantly couple to localized charge states and be used to modulate their charge carrier dynamics in the ultrafast time scale.^6^ Such photonic control offers a potential tool to control carrier transport on demand.

In this work, we take advantage of the current-sensitive optical modulation pump-push-photocurrent (PPPC) approach and report a methodology capable of directly tracking the effect of charge carrier dynamics on the PEC photocurrent output. Using BiVO_4_ photoanodes as an example, we demonstrate that oxygen vacancy states act as recombination centers after carrier trapping even at the interface, where the electric field is maximized. Moreover, we also show that reactivation of the trapped carriers with IR light produced additional photocurrent and is beneficial to PEC performance. By observing carrier detrapping under different working conditions, we concluded that extra bias is needed to achieve charge carrier separation and thus better quantum efficiency after carrier trapping occurs in vacancy states. The PPPC method allows us to expose charge localization pathways, validate previous mechanistic models, and provide the first direct proof that charge localization depends strongly on the electric field within the device. Our results also demonstrate that IR optical control is not a curiosity for the spectroscopist but can be used to exert dynamic control of localized charges and alert PEC photocurrent under working conditions.

## Results and Discussion

### BiVO_4_ Photoanode Characterization

This study was carried out on BiVO_4_ photoanodes in water-splitting PEC cells. The photoanodes were composed of ∼350 nm thick BiVO_4_ films fabricated by metalorganic decomposition method.^48,49^ XRD patterns (Figure S1a) confirm the material possessed the monoclinic scheelite structure, which is known to show the highest water splitting activity in this material.^50^ The PEC water oxidation performance of our BiVO_4_ photoanodes was measured in a three-electrode configuration (Figure S2a), and the photoanode exhibits a 0.6 V_RHE_ onset potential in agreement with results from the literature.^47,49^ The *J–V* curve measured in a two-electrode configuration from the pulsed laser light used in our PPPC experiments is shown in Figure S2b. The curve shows a trend and onset potential similar to those of the *J–V* measured by monochromatic blue light in the same two-electrode configuration. Further on, we transferred potentials under two electrodes to potential versus RHE according to the comparison between Figure S2a and Figure S2b. The converted values are aiming to provide a reference on how large the applied potential is compared to the onset potential, rather than give a precise corresponding value between three and two electrode measurements. The photocurrent measured by the lock-in amplifier is lower compared to the photocurrent measured by the potentiostat, as the lock-in uses fast modulations and is only sensitive to the fast response processes from the cell (Figure S3a shows how modulated photocurrent increases as modulation frequency).

**Figure 1:**
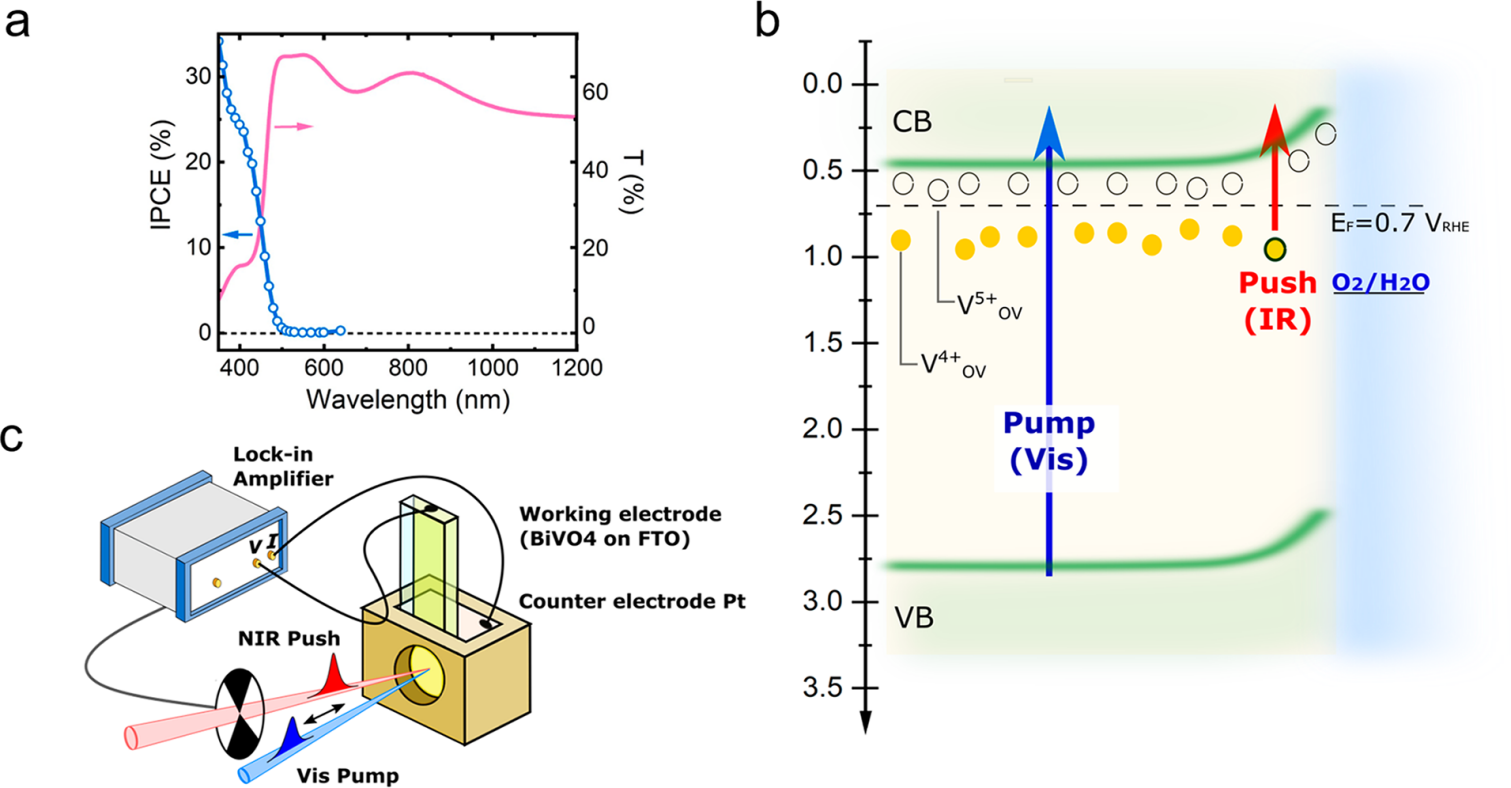
(a) Left axis: IPCE of BiVO_4_ photoanode under 1.23 V_RHE_, with three-electrode electrochemical cell, under back-side illumination. Right axis: UV–vis transmittance spectrum of 350 nm thick BiVO_4_ film. (b) Simplified band diagram of BiVO_4_ under 0.7 V_RHE_ bias. *E*_F_ is the Fermi level of BiVO_4_ (under the applied bias condition). V^4+^ and V^5+^ (circles) are filled and available oxygen vacancy states, respectively. The straight arrows indicate the optical transitions induced by pump (blue) and push (red) pulses. (c) Illustration of the Pump-Push-Photocurrent (PPPC) spectroscopy setup. In all experiments, the BiVO_4_ photoelectrode was excited from the back side in 0.1 M phosphate buffer (pH 7).

Figure 1a shows the transmittance spectrum of the film across the UV–NIR region. We observe a prominent absorption at ∼425 nm, which is associated with the band-to-band electronic transition characteristic of monoclinic scheelite BiVO_4_.^50,51^ The spectrum also shows a broad, featureless band spanning the near-IR, which we attribute to transitions from sub-bandgap states to the conduction band, as observed in other metal oxides.^7^Figure 1a also displays the incident photon-to-current efficiency (IPCE) measured at 1.23 V_RHE_ in a three-electrode configuration with back side illumination. We observe an onset at ∼500 nm, which is in agreement with the absorption edge seen in the UV–visible spectrum. As expected, the IPCE at 400 nm with back illumination is ∼25%, in agreement with reported efficiencies for this material.^51,52^ Critically, we observe that the photocurrent at wavelength >500 nm drops by more than 2 orders of magnitude, confirming that no substantial contribution to the solar water splitting process occurs from light absorption by sub-bandgap state.

### Optical Control of Photocurrent Approach

Figure 1b shows a simplified band diagram of BiVO_4_ to help contextualize our optical control strategy. Vacancy-associated states result in a distribution of V^4+^/V^5+^ states below the conduction band.^47,53^ Reduced vacancies can be formally considered as V^4+^ states, while oxidized states can be considered V^5+^ states. The diagram depicts a flat band potential of 0.35 V_RHE_ (previously measured in photoanodes prepared with the same method),^47^ as well as the emergence of band bending and buildup of a space charge layer (SCL) due to biasing with 0.7 V_RHE_ (0.3 V_Pt_). A previous study utilizing XPS analysis reported an oxygen vacancy ratio of 1.68% in BiVO_4_ prepared by using the same method. This estimation was based on calculation of the peak ratio between V^4+^ and V^5+^ species in the XPS spectra. In many metal oxides like BiVO_4_, oxygen vacancies typically act as the source of charge in the equilibration with the electrolyte leading to the accumulation of V^5+^ states in the SCL.^54^ Some measurements in this work are under short circuit conditions; this corresponds effectively to 0.6 V_RHE_, which lies positive of the flat-band potential. We thus consider that band bending is present under all conditions studied herein. Figure 1c shows our optical control strategy based on a pump-push-photocurrent (PPPC) experiment. For steady-state PPPC, the PEC is illuminated by two continuous-wave (CW) lasers: (i) a 405 nm *pump* (vis) which promotes electrons from the valence to conduction bands and (ii) a 980 nm *push* (IR) which modulates subgap states. For time-resolved experiments, we use short laser pulses of (i) 100 fs/400 nm as the *pump* and a (ii) 1 ns/1064 nm IR as the *push* with a repetition rate of 4 kHz. The time delay between pulses is controlled with a delay generator with the accuracy of 8 ns. The PPPC measurements in this work are conducted under back side illumination to facilitates electron transport after IR reactivation.

To simplify photocurrent detection, a two-electrode cell is employed by using platinum as the counter electrode. The BiVO_4_ remains in contact with the electrolyte during the measurement, thereby enabling an *in situ* measurement of the photoexcited carrier dynamics following both pump and push excitation. The currents upon visible-pump-light irradiation (*J*_vis_) and IR-push-light excitation (d*J*_IR_) are detected using a lock-in amplifier under identical conditions by using a modulating chopper in either beam. Visible-pump photocurrent *J*_vis_ originates from exciting carriers from the valence band to the conduction band followed by their transport and extraction to the external circuit. Likewise, d*J*_IR_ relates exclusively to subgap IR excitation of localized carriers increasing photocurrent generation (see arrows in Figure 1b). In particular, ultrafast optical studies have shown that IR excitation can modulate oxygen-vacancy states in the time domain of our experiments.^47^

### Optical Control of Oxygen Vacancies under Steady-State Illumination

**Figure 2:**
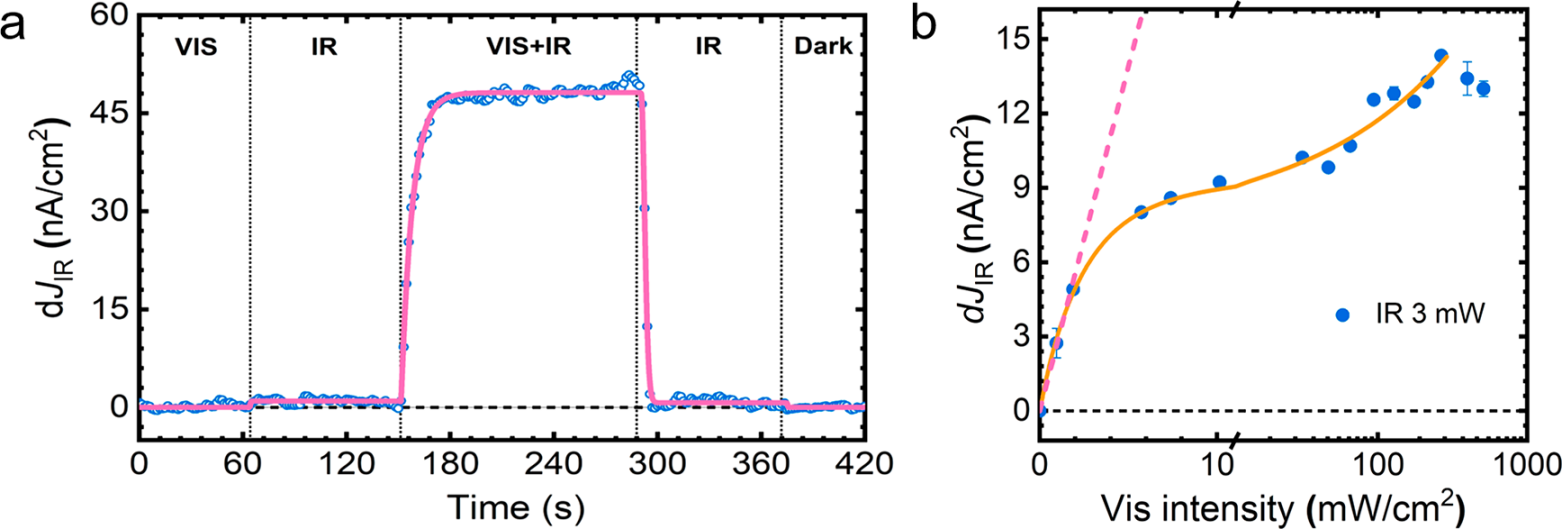
(a) Light induced photocurrent from continuous 405 nm visible (6 mW/cm^2^) and 980 nm IR (0.26 W/cm^2^) under 0.7 V_RHE_. (b) Continuous laser diode excitation intensity effect: light induced photocurrent under fixed IR (0.26 W/cm^2^) with different visible pump intensities under no external applied bias. In all experiments, the BiVO_4_ photoelectrode was excited from the back side in 0.1 M phosphate buffer (pH 7).

First, we evaluated the feasibility of the PPPC approach to modulate the performance of an operando PEC cell. Figure 2a shows the quasi-steady-state pump-push photocurrent (PPPC) measurements at 0.7 V_RHE_ (i.e., after the onset). We note that this representation shows only current values measured with the optical chopper on the IR-push beam. Consequently, the visible-pump-induced IR-push photocurrent appears as a zero baseline in this plot (for reference, the visible-pump current was 0.04 mA/cm^2^ when the visible path was modulated).

As shown in the figure, we observe no current in the absence of illumination. Similarly, when only IR-push light is present, we observe a very moderate d*J*_IR_, which is associated with the excitation of filled vacancy states (V^4+^) near the conduction band and their extraction through an external circuit. As photocurrent generation in PEC takes place primarily within the SCL and vacancy states are depleted in the region,^55^ it is not surprising that the IR-push current is so low in the absence of visible light. We attribute d*J*_IR_ signal dominantly to the reactivation of trapped electrons in the space charge layer since the photocurrent difference detection using a lock-in amplifier is only sensitive to the “effective” reactivation processes, followed by efficient extraction of carriers. While trapped electrons in the bulk region (near the FTO side) can be detrapped by IR light, they are likely to recombine again.

In contrast, when the cell is simultaneously irradiated with both visible-pump and IR-push light, the d*J*_*IR*_ response is significantly enhanced. This indicates that following band gap irradiation, a larger population of subgap states can be excited by the push beam. Notably, this population was not present in the dark and is thus generated by the visible-pump beam. Building on previous spectroscopy studies,^47^ we propose that pump illumination generates a distribution of mobile electrons in the conduction band, some of which become trapped in empty vacancy states within the SCL, reducing them from V^5+^ to V^4+^. These trapped carriers can be subsequently re-excited by the IR-push, becoming available for extraction and increasing the photocurrent d*J*_*IR*_.

Importantly, without the availability of IR-push light, charges trapped in subgap states are likely to recombine and do not contribute to photocurrent generation, and the d*J*_IR_ represents the population of trapped carriers that are effectively saved and released through the reactivation process facilitated by the IR light. To our knowledge, the results in Figure 2a provide the first demonstration that targeted optical excitation of defect states can be used as a tool not only to change the ultrafast optical response of the sample but also to modulate charge transport and photocurrent in operating PEC cells.

Figure 2b shows the dependence of the IR-Push photocurrent, d*J*_IR_, on the CW-visible-pump intensity (see Figure S3b for IR-push intensity dependence). The d*J*_IR_ photocurrent increases linearly with low visible-pump power (from ∼0 to 2 mW cm^–2^) but starts to plateau at higher pump powers (from ∼10 to 100 mW cm^–2^). This behavior indicates that at high visible pump fluences, a smaller fraction of free carriers is available for trapping and eventual detrapping. This agrees with the higher bimolecular recombination yields as observed in ultrafast optical studies and intensity dependence of CW Vis pump induced *J*_vis_ (Figure S3d), indicating that this recombination pathway is a viable photocurrent loss path in operando cells.

In the next experiments, we limit our measurement to the linear region where trapping is the dominant loss pathway. We suggest that detecting IR-push photocurrent (d*J*_IR_) provides a reliable indicator of the average concentration of electrons trapped in vacancy states within SCL. Calculating d*J*_IR_/*J*_vis_, in this case, reflects the ratio of trapped and free carrier concentrations within SCL.^56^ We have previously shown that for organic photovoltaic devices d*J*_IR_/*J*_vis_ does not depend on the bias- and intensity-dependent carrier extraction.^56^ Therefore, using d*J*_IR_/*J*_vis_ in the analysis offers a more direct comparison of the results measured under different experimental conditions.

### Time-Resolved Observation of Carrier Localization Dynamics

**Figure 3:**
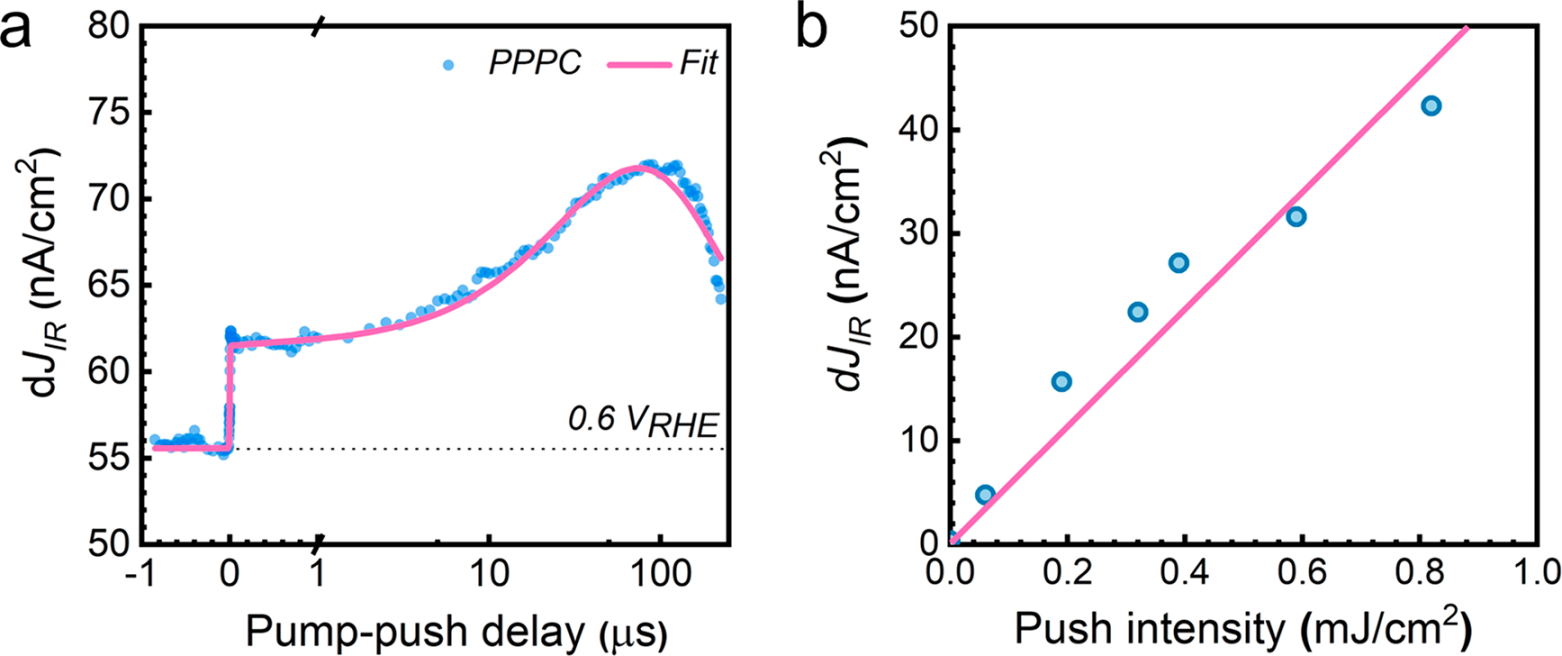
(a) Push-induced PPPC carrier dynamics under short-circuit conditions where recombination dominates. The BiVO_4_ film was excited with a vis 405 nm (1.3 μJ cm^–2^) pump and an IR 1064 nm (0.3 mJ cm^–2^) push from the backside of the sample in 0.1 M phosphate buffer (ph 7). (b) Linear fit of the push intensity-dependence of pump-push-photocurrent at 80 μs. The sample was excited with a vis 405 nm (1.3 μJ cm^–2^) pump and an IR 1064 nm push. All experiments are measured in a designed 2-electrode PEC cell unless specified.

Having observed that the optical control of localized states can enhance the photocurrent of the PEC cell, we next evaluate the underlying mechanism. To this goal, we probe carrier dynamics in the nanosecond to millisecond time domain. This is a reasonable time window, as the mobile electron extraction of BiVO_4_ typically occurs on the μs to ms time scale.^57^Figure 3a shows the time-resolved change in IR-push current under short-circuit conditions. At negative times (before the visible pump interacts with the sample), we observe a small background d*J*_IR_ photocurrent. We attribute this background photocurrent to the IR activation of long-lived electrons within the SCL which have lifetimes >250 μs and which were generated by the preceding pump pulses. The origin of this signal is therefore identical to signals observed in steady-state PPPC.

At *t* = 0, when the visible-pump and IR-push pulses coincide in time, a rise in the positive d*J*_IR_ signal is observed. The resolution-limited sharp rise indicates that some charge carriers become trapped in subgap states at the SCL faster than the 8 ns time resolution of our measurement in agreement with previous studies.^58,59^ It is worth noting that the contribution of self-trapped carriers through polaron localization to V sites is highly likely to be involved in the d*J*_IR_ signal at the ultrafast time scale.^60,61^ As shown in Figure S4, the PPPC carrier dynamics from femtoseconds to hundreds of picoseconds exhibit an additional fast decay component (∼5 ps) preceding the increase in signal (∼50 ps) associated with trapping in oxygen vacancy states, which is likely related to the reactivation of self-trapped carriers. Interestingly, after the initial rise, the d*J*_IR_ signal exhibits additional delayed growth until ∼100 μs indicative of a population of excited electrons that trap in oxygen vacancy associated states on such timescales. This slow behavior contrast with previous time-resolved optical measurements which suggested electron–hole recombination dominated after fast charge trapping.^23^ Instead, our current-sensitive measurements provide the first operando demonstration that trapping extends over 100 μs. Subsequently, after 100 μs, the current change decreases reflecting a decrease in the population of re-excitable trap carriers. The fs PPPC dynamics (Figure S4) indicates the growth of ns PPPC signal starts from ∼50 ps, which agrees with earlier studies revealing ps-time scale dynamics after IR excitation.^47^ The ultrafast time scale of the response, the complex shape, and bias dependence of the PPPC kinetic provide strong evidence that the observed signals do not originate from sample heating by IR light.

The complete dynamics of the trapped carrier population can be fit with a combination of instant and delayed exponential growth models multiplied by a single exponential decay, reflecting the recombination process. The fitted curve shown in Figure 3a represents convolutions of these kinetics with a Gaussian response function (Equation S2 in the Supporting Information). Based on the slow carrier mobility in BiVO_4_,^62^ we attribute the multiphasic PPPC dynamics to different trapping mechanisms. At early times (<1 μs) trapping occurs locally (time constant τ0 shown in Table S1), while at longer times (τ1 = 62 μs) trapping is assisted by charge transport, likely due to thermally activated hopping.^63^ At longer times, recombination occurs with a time constant of τ2 = 135 μs. As shown in Figure 3b, we found that the PPPC signal scales approximately linearly with the IR push intensity <0.8 mJ cm^–2^, indicating that photophysics at this illumination power is similar to that under solar illumination conditions. A control nanosecond PPPC experiment is conducted on a thinner BiVO_4_ sample (∼ 175 nm thick). As shown in Figure S5, both the 350 and 175 nm samples exhibited nearly identical ns PPPC dynamics after normalization. This observation suggests that the hole transport length is not limiting the dynamics results observed in the photocurrent measurements herein.

### Illumination and Voltage Dependence of Charge Localization and Re-excitation

**Figure 4:**
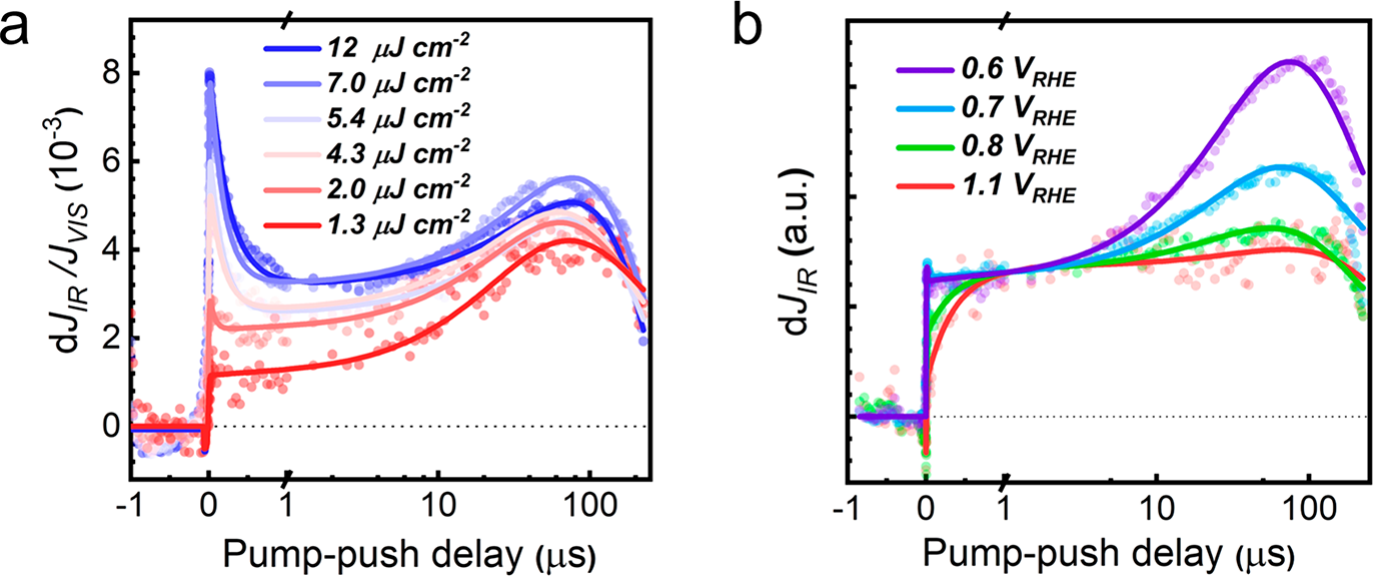
(a) Trap carrier dynamics of BiVO_4_ under different pump intensity, data normalized to the photocurrent under only visible light at 0.6 V_RHE_. (b) Trap carrier dynamics of BiVO_4_ under different bias conditions. For clarity, the data is normalized to the value at 1 μs d*J*_*IR*_ of 0.6 V_RHE_. The IR push fluence for (a) is 0.3 mJ cm^–2^, and the fluences for (b) are 1.3 μJ cm^–2^ vis pump and 0.3 mJ cm^–2^ IR push. All experiments are measured in two-electrode PEC cells in 0.1 M phosphate buffer (pH7) under 0.6 V_RHE_.

Figure 4a shows the ratio between the IR detrapped and mobile carrier concentrations (d*J*_IR_/*J*_vis_) as a function of the visible pump fluence at a fixed, 0.6 V_RHE_, applied bias. Following the prompt signal increase at *t* = 0 we observe that at low fluence, the signal is initially flat and subsequently grows gradually after 1 μs. In contrast, at high pump fluences we observe the emergence of a fast-decaying (<1 μs) component. The amplitude of the signal increases with fluence while the dynamics in this region stay roughly the same. The estimated time constants of the fast decay (τ0) and slow growth (τ1) are presented in Table S1.

We attribute the fast ∼200 ns decay to bimolecular recombination at high carrier concentrations, reducing the population of states that can be controlled by the push. Bimolecular recombination is expected to be strong at low applied bias as both holes and electrons are distributed relatively homogeneously throughout the BiVO_4_ film. In addition to the fast decay, we also observe an instantaneous response in PPPC data at *t* = 0 which is noticeable at all pump powers between 1.3 and 12 μJ/cm^2^ (Figure S6a). This component is within our time ∼20 ns time resolution, and its presence agrees with previous transient absorption observation of fast hole trapping,^47^ which reduces the concentration trapped electron monitored in PPPC measurements.

The operating conditions of PECs may involve the application of an external bias, which substantially affects charge carrier dynamics.^58^ Under applied bias, the V^4+^/V^5+^ distribution changes due to the field redistribution across the BiVO_4_ film and the spatial extension of the SCL. Both of these phenomena strongly affect carrier transport. Figure 4b shows the dependence of the IR-push induced current dynamics on the external applied potential (full data set presented in Figure S7). The PPPC trace under 0.6 V_RHE_ is the lowest pump intensity data set in Figure 4a. The d*J*_IR_ values at different bias are normalized according to photocurrent at 1 μs under the 0.6 V_RHE_ condition for a better comparison of the dynamics.

The evolution of PPPC dynamics with bias is complex and with two key features: (i) with increasing bias, the early dynamics change from a step-like shape to a gradual ∼200 ns growth, implying a slower/weaker local electron trapping, and (ii) the slow transport-assisted ∼10 μs trapping component gradually decreases, as the bias is increased from 0.6 to 1.1 V_RHE_. Such a major decrease in the trapping component likely comes from a combination of effects, including a widening of the SCL. The time constants of both the growth and decay dynamics, summarized in Table S2, indicate that both processes slow down with increasing bias.

**Figure 5:**
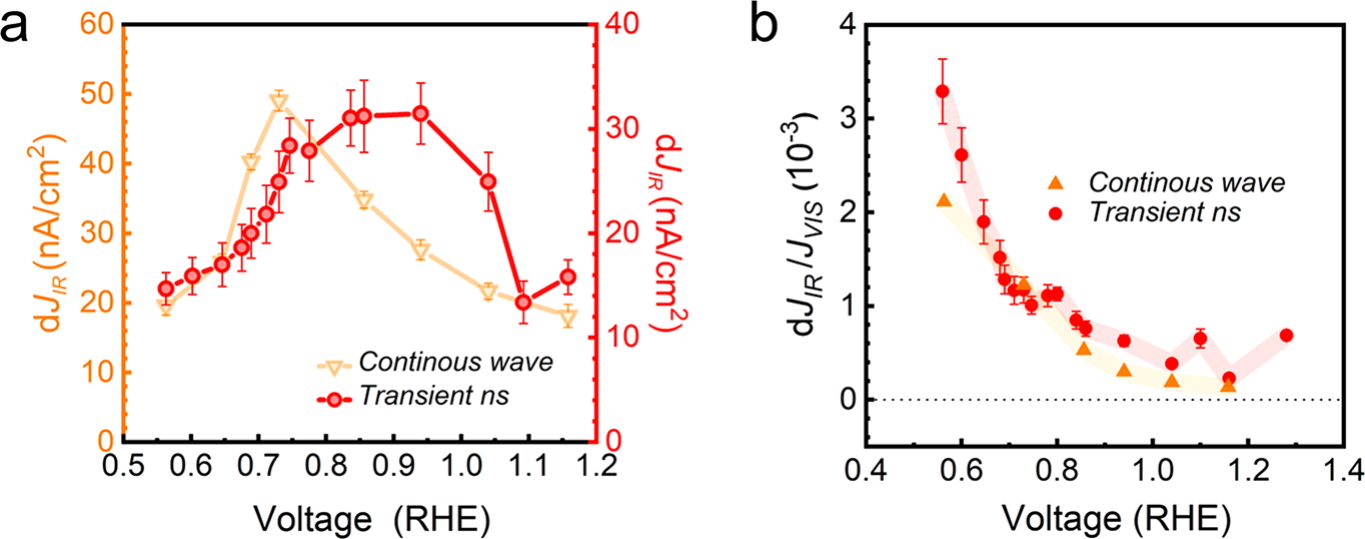
(a) Average PPPC d*J*_*I*__R_ amplitude under continuous wave laser conditions and transient conditions as a function of the external applied bias. The transient condition photocurrent average is calculated from data across the 80–200 μs window presented in Figure S7. The continuous wave PPPC is conducted under bias with a 405 nm Pump (6 mW/cm^2^) and 980 nm Push light (0.13 W/cm^2^). (b) Influence of the applied bias on the ratio between the trap carrier and the band carrier. Raw data presented in Figure S7b, the transient condition photocurrent average is obtained from the data across the 80–200 μs window.

Both the shape and amplitude of the PPPC kinetics are affected by the external field. Figure 5 shows the evolution of both the IR-push current (d*J*_*IR*_) and the ratio of trapped-to-free carriers (d*J*_IR_/*J*_vis_) as a function of the applied bias (raw data are shown in Figure S7). Initially, at low biases (*V* < 0.94 V_RHE_), which are sufficient to create an SCL with ∼600 mV positive of the flat band potential, the amplitude of d*J*_IR_ increases (Figure 5a) indicating the trapping of more carriers and their subsequent re-excitation. This trend then breaks above 0.94 V_RHE_, when the signal decreases, indicating less carrier trapping. This behavior suggests the potential gradient within the SCL becomes sufficiently strong to remove electron carriers out of traps and directly reduce current losses. In contrast, we observe that the ratio of trapped-to-free carriers (d*J*_IR_/*J*_vis_) decreases monotonically with increasing bias (Figure 5b), reflecting a steady decrease in the relative number of trapped carriers, and respectively a smaller contribution of detrapped carriers to device photocurrents.

### Voltage Dependence of Charge Localization under Steady-State Illumination

Figure 5 also shows the IR push current dependence under steady state conditions as a function of the applied potential. Interestingly we observe the same trend as in the time-resolved measurements. Namely, at low applied bias, trapping dominates and becomes increasingly detrimental until 0.72 V_RHE_ after which the field strength is sufficient to start delocalizing electrons and promote charge extraction.

Our photocurrent measurements provide direct proof that the significantly high number of trapped electron carriers observed at low bias is likely a major cause of low device performance under these conditions. Importantly, we observe that trapping is not only under pulsed conditions, typically used in time-resolved optical studies, but also present under steady state conditions used in operation. While multiple synthetic and thermal strategies have been developed to control trapping, we propose that optical control of this carrier with IR light can provide a tool to circumvent this loss pathway in a dynamical way, as shown in Figure 2a. We note that in our experimental configuration current gains are still limited. However, we argue that photonic control could offer a way to harvest the IR spectrum and control the operation on demand. This could enable, for example, rapid and reversible performance adjustments to solar intensity conditions and would benefit from developments in photonics and light management strategies already used in other technologies.

### Device-Performance and Trap-Activation Model

**Figure 6:**
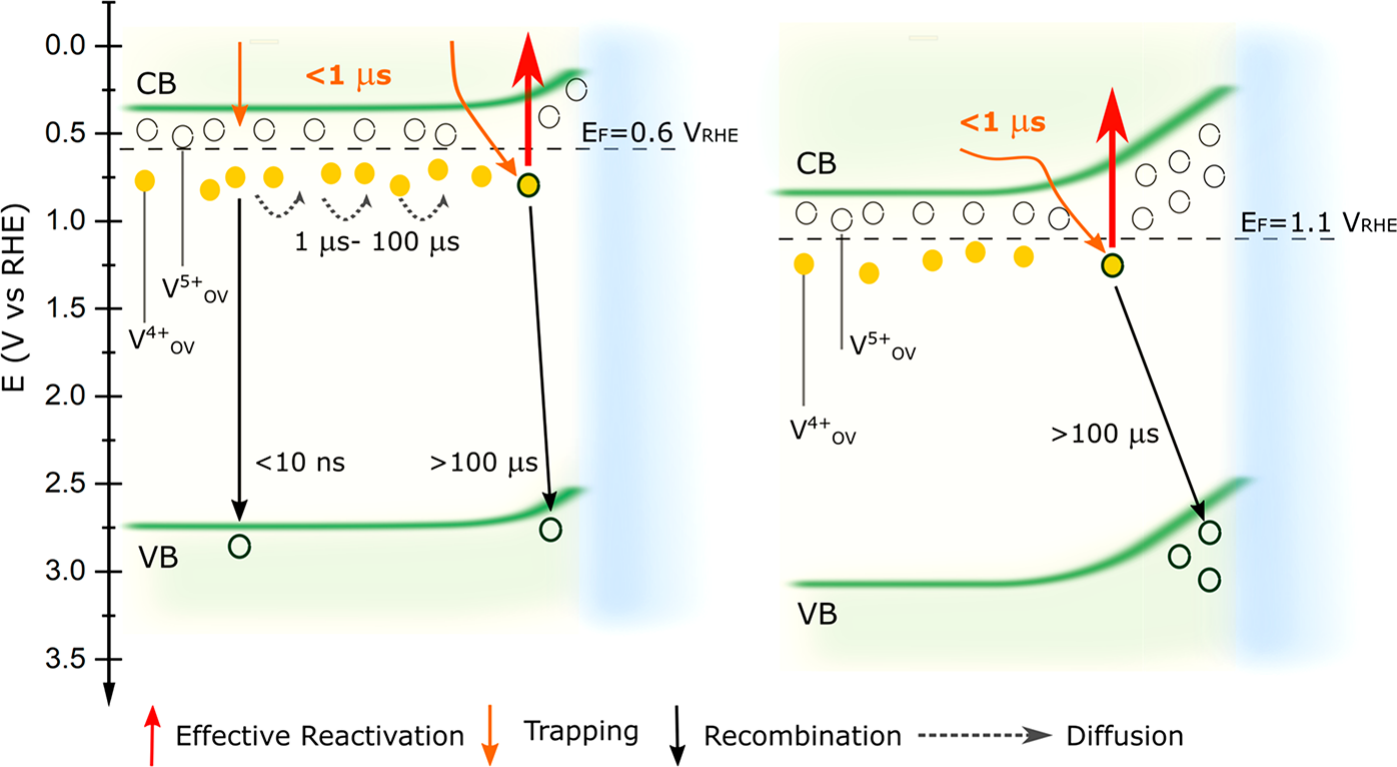
Simplified model of the elementary processes that occur in a BiVO_4_ photoanode under short-circuit (0.6 V_RHE_) and 1.1 V_RHE_ (1.2 V_Pt_) anodic bias conditions.

Figure 6 presents a qualitative summary that consolidates the charge carrier dynamics observed via PPPC measurements. Under the short-circuit conditions, in contact with the electrolyte, electrons regenerated within SCL fall into oxygen vacancy states rapidly (<10 ns). Due to the electric field in SCL, electrons are trapped until they recombine or are activated by IR light. Under normal operating conditions, most electrons trapped in the vacancy states in the bulk do not contribute to the photocurrent.^47^ This likely also applies to electrons released by IR activation and agrees with the low activation when the cell is illuminated only with CW-IR-push light (Figure 2a).

However, the electrons generated at the bulk/SCL interface have a finite probability of diffusing into the SCL and becoming trapped. From there they can be re-excited with IR light and produce d*J*_IR_. We propose this process manifests as a transport-assisted slow 60 μs rise in our transient data (Figure 3a). Subsequent recombination of these carriers leads to a PPPC decay within 100 μs. We propose that most of the effective trap carrier reactivation happens near the bulk/SCL interface, as the internal electric field will help carrier separation and extraction.

In contrast, at a high external bias of 1.1 V_RHE_, the field is much stronger. This assists the hopping of electrons localized in vacancy states making them effectively more mobile, facilitating extraction, and suppressing trapping. Indeed, we observe an increase of fast rise τ3 in the buildup of the PPPC signal due to the reduced electron trapping. The diffusion of electrons from bulk to SCL goes against the applied field and, thus, leads to an obvious increase in slow trapping time τ4. As expected, at late times, recombination through vacancy states is suppressed by the internal electric field, leading to a longer trapped carrier lifetime.^1^ Note that in this model, we assume that carrier diffusion becomes negligible compared to their drift under high bias.

In summary, here we report the operando photocurrent-resolved trap carrier dynamics in BiVO_4_ photoanodes. Our data provides device-relevant validation that vacancy states in BiVO_4_ serve as traps for electrons after ultrabandgap excitation. The results indicate that oxygen-vacancy-associated states determine the effective carrier concentration available for extraction in BiVO_4_ and thus the final PEC performance. Critically, we show that it is possible to control the population of photolocalized charges through the use of targeted IR light, and that this process is not mediated by sample heating. The photon-induced modulation allows control of charge carrier deactivation processes in a dynamic manner and results in direct changes in the charge extraction in operando PEC conditions. Our results show that time-resolved photocurrent-sensitive measurements provide a valuable tool to assess effective loss pathways in PEC cells, complementing and validating traditional time-resolved optical measurements. Moreover, we argue that photonic control of operando PEC cells can provide a new degree of freedom to boost and tune performance on demand.

## Methods

### Preparation of BiVO_4_

All films were spin coated on TEC 15 FTO substrates from Pilkington NSG. All chemicals were from Sigma-Aldrich unless specified. The FTO substrates were washed with detergent, deionized water, and isopropanol (IPA), respectively. The substrates were then calcined at 500 °C for 30 min before applying the BiVO_4_ coating. BiVO_4_ was prepared through a modified metal–organic decomposition procedure, reported elsewhere.^48,49^ Bismuth nitrate (Bi(NO_3_)_3_) (98%) and vanadyl acetylacetonate (98%) precursor solutions were prepared separately. 0.07275 g (200 mM) Bi(NO_3_)_3_ was dissolved in 0.75 mL of acetic acid, and 0.0384 g (30 mM) vanadyl acetylacetonate was dissolved in 2.5 mL of acetylacetone. The two solutions were mixed and stirred at room temperature for 30 min to form a sol–gel. The sol–gel mixture was then deposited on FTO by spin-coating. 50 μL of the sol–gel solution were used (with a rotation speed of 1000 rpm and coating for 20 s) for the deposition of each layer. After deposition of every layer, the substrates were calcined in the preheated oven at 450 °C for 10 min. Depending on the required thickness, the process can be repeated several times. For this work, the deposition process was repeated 14 times (i.e., 14 layers were coated). After the deposition of the final layer, the film was further calcined at 450 °C overnight.

### Photoelectrochemical Cell and Characterization

All photoelectrochemical (PEC) measurements were performed in a specially designed homemade cell. The cell was designed to reduce the noise measured by PPPC and allow for the detection of low-level photocurrent induced by trapped carriers. All PPPC measurements were carried out in a two-electrode configuration to allow lock-in detection. The cell consists of three chambers that are linked to each other. The BiVO_4_ working electrode was contacted with the electrolyte in the main chamber through a 1 mm hole, which is designed to reduce the dark current (and increase the overall signal-to-noise ratio) detected by a lock-in amplifier (Zurich). The counter electrode (Pt wire) was immersed in the electrolyte of the second chamber. Visible and IR light was passed through the 1 mm hole and illuminated the BiVO_4_ through the back of the photoelectrode (unless specified). All PEC measurements were carried out in pH 7 phosphate buffer (0.1 M).

Standard PEC characterization was performed in a three-electrode configuration with saturated KCl Ag/AgCl (Metrohm) as the reference electrode. For Linear Sweep Voltammetry (LSV) and incident photon-to-current conversion efficiency (IPCE) measurements, a monochromator (OBB-2001, Photon Technology International) coupled to a 75 W Xe lamp (USHIO) was used as the light source, and the potential was set by an Autolab potentiostat (PGSTAT 12, Metrohm). The data were recorded by using the Nova software. The *J*–*V* curves were measured under 4 mW/cm^2^, with a 10 mV s^–1^ scan rate. The intensity of the simulated sun spectrum with a monochromator (shown in Figure S2c) was measured with an optical power meter (PM 100, Thorlabs) equipped with a silicon photodiode (S120UV, Thorlabs). The IPCE was calculated using the following equation:
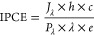
where *J*_λ_ (mA cm^–2^) is the photocurrent density under a single wavelength, *h* is Planck’s constant, *c* is the speed of light, *P*_λ_ (mW cm^–2^) is the power intensity of the monochromatic light at a given wavelength, λ(nm), and *e* is the charge of an electron.

The potential conversion between two (vs Pt) and three electrode systems are performed by corresponding the potentials in LSV measurements with the same photocurrent, which are the data shown in Figure S2a and b. The LSV measurements are measured on the same sample, equipment, and electrolyte in 1 day.

### Pump-Push-Photocurrent (PPPC) Spectroscopy

A continuous wave PPPC and a nanosecond to millisecond PPPC setup (Figure 1c) was built to measure the photocurrent changes occurring during charge trapping and detrapping. The “pump” refers to visible light, which generates electrons and holes that are used to drive the water-splitting chemical reaction. The “push” is IR light that can re-excite electrons in trap states. In continuous wave PPPC, the pump and push light are a collimated laser diode module from Thorlabs of 405 nm (CPS405) and 980 nm (CPS980) respectively. In nanosecond-to-μs PPPC, the 800 nm 4 kHz Ti:sapphire regenerative amplifier (Astrella, Coherent) produced ∼35 fs pulses that were used to seed a β-barium borate (BBO) doubling crystal. BBO produced 400 nm light by second harmonic generation, which was used as a Pump light. The 1064 nm push light was generated with an INNOLAS Nd:YAG Laser (P1725). In both setups, the pump and push beams were focused onto an ∼1 mm diameter spot on the electrochemical cell. Both the pump and the push were modulated by an optical chopper system (Thorlabs MC2000B). The two lights are combined after a beam combiner, focused to the sample position, and illuminated on BiVO_4_ film through the hole on the electrochemical cell. A lock-in amplifier (Zurich MFLI) detects the photocurrent synchronized with the chopper modulation frequency. The lock-in amplifier also works as a power supply to add anodic bias to the BiVO_4_ photoelectrode. The continuous and transient PPPC setup allows the detection of the pA level current. The low sensitivity is important for the detection of IR-induced current under low-intensity illumination, which is typical of natural solar irradiation.

### UV–vis Absorption Spectroscopy

The absorption spectra of the BiVO_4_ films were characterized with a PerkinElmer UV–vis spectrometer 45 (Lambda 25) with a slit width of 5 nm. Transmittance data are measured between 350 and 1200 nm.

### XRD

X-ray diffraction (XRD) patterns were measured with a modified Bruker-Axs D2 diffractometer with parallel beam optics equipped with a PSD LinxEye silicon strip detector. The Bruker XRD uses a Cu source for X-ray generation (*V* = 40 kV, *I* = 30 mA), with Cu K_α1_ (λ = 1.54056 Å) and Cu K_α2_ radiation (λ = 1.54439 Å) emitted with an intensity ratio of 2:1. The sample was measured in the angular range between 10 ≤ 2θ° and 60° with a step size of 0.05°, with the incident beam kept at 1°.

## Supporting Information Available

Materials Characterization; Electrochemical and Photoelectrochemical measurements; Modulation Freq, intensity and bias dependence of CW PPPC measurements; Femtosecond PPPC measurements; Control ns PPPC measurements of different thickness BiVO_4_; Intensity and bias dependence of transient PPPC measurements; Fitting equations of PPPC data; Fitting parameters of CW and transient PPPC data. (PDF)
