# Supplementary material for: Operando IR Optical Control of Localized Charge Carriers in BiVO4 Photoanodes
Source: J Am Chem Soc. 2023 Aug 1;145(32):17700–9. doi: 10.1021/jacs.3c04287 (PMC10436276; doi:10.1021/jacs.3c04287)
Supplement: Supplementary file 1 — ja3c04287_si_001.pdf [file ja3c04287_si_001.pdf]

Supporting Information for

Operando IR Optical Control of Localized Charge Carriers in $BiVO_4$ Photoanodes

Zhu Meng, [a] ***Ernest Pastor,*** [b*] ***Shababa Selim,*** [a] ***Haoqing Ning,*** [a] ***Marios Maimaris,*** [a] ***Andreas Kafizas,*** [a,c] ***James R. Durrant,*** [a] ***and Artem A. Bakulin*** [a*]

[a] *Department of Chemistry and Centre for Processible Electronics, Imperial College London, London W12 0BZ, United Kingdom*
[b] *IPR–Institut de Physique de Rennes, CNRS-Centre national de la recherche scientifique, UMR 6251 Université de Rennes, 35000 Rennes, France*
[c] *London Centre for Nanotechnology, Imperial College London, London SW7 2BP, United Kingdom*

******Corresponding Authors:* a.bakulin@imperial.ac.uk, ernest.pastor@univ-rennes.fr

Table of Contents

**Section**	**Page number**
Materials Characterization	S2
Electrochemical and Photoelectrochemical measurements	S2
Modulation Freq, intensity and bias dependence of CW PPPC measurements	S3
Femtosecond PPPC measurements	S4
Control ns PPPC measurements of different thickness $BiVO_4$	S4
Intensity and bias dependence of transient PPPC measurements	S5
Fitting equations of CW and transient PPPC data	S6
Fitting parameters of transient PPPC data	S6

1. Supplementary figures:

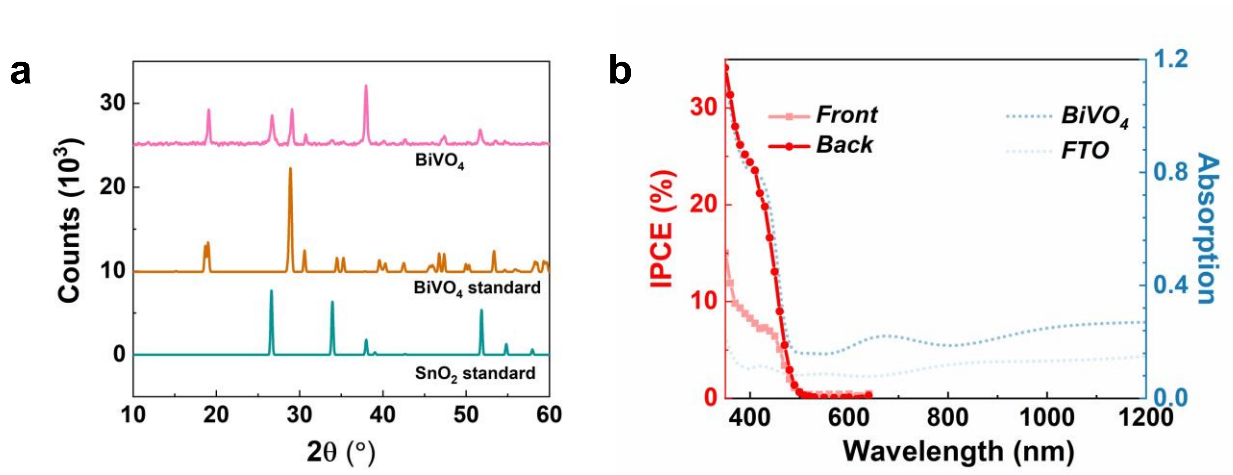

Fig. S1 (a) XRD of $BiVO_4$ film. A typical XRD pattern of the $BiVO_4$ photoelectrode grown on FTO (F: SnO_2 glass) shown alongside reference standards (monoclinic scheelite $BiVO_4$ and tetragonal cassiterite SnO_2). **(b)** IPCE under back and front illumination at 1.23 VRHE and UV-vis of the $BiVO_4$ photoelectrode and FTO glass.

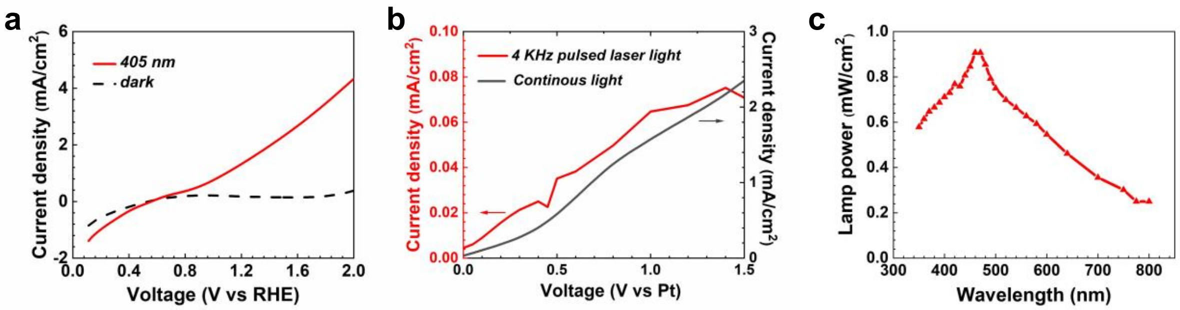

Fig. S2 (a) Linear sweep voltammograms (LSV) of the $BiVO_4$ photoelectrode in the three-electrode configuration. The photoelectrode in 0.1 M phosphate buffer is illuminated from the back with 405 nm (~4 mW/cm^2). **(b)** Photocurrent of $BiVO_4$ under bias in the two-electrodes configuration. Black line was measured by LSV under monochromatic blue light (400 nm, ~4 mW/cm^2), red line was measured by PPPC pulsed 405 nm light (46.5 mW/cm^2), respectively. **(c)** Power output of the Xe lamp at various wavelengths set by the monochromator for IPCE measurements.

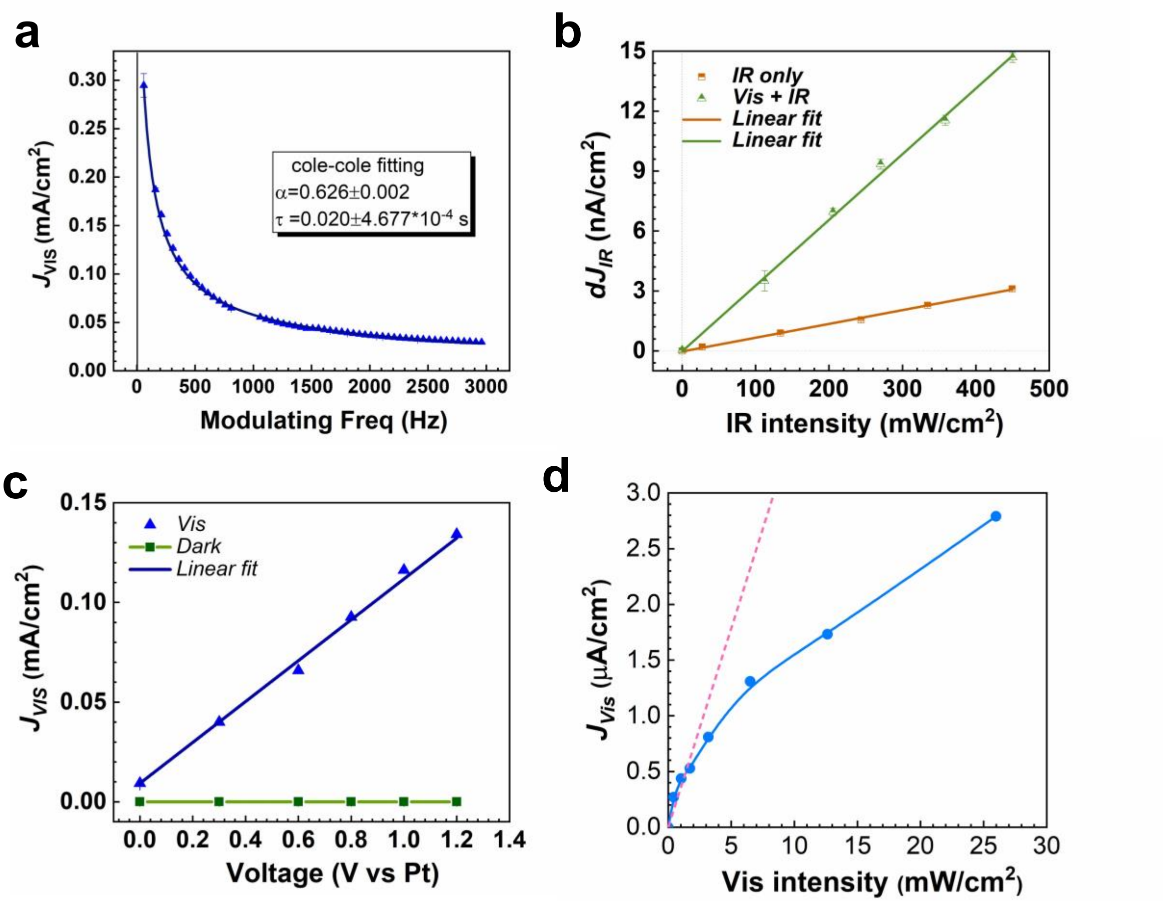

Fig. S3 (a) Dependence of Vis light induced photocurrent on modulation frequency, Vis intensity: 9 mW/cm^2, Cole-Cole fitting equation shown in Eq. S1. **(b)** Intensity dependence of continuous wave dJ_{IR} under only IR push (980 nm) and both Vis (450 nm, 40.8 mW/cm^2) pump and IR push. **(c)** Bias dependence of continuous wave J_{vis} with Vis pump only (12.7 mW/ cm^2). **(d)** J_{vis} with different pump intensity under no external bias. The $BiVO_4$ photoelectrode was excited from the back side in 0.1 M phosphate buffer (pH 7).

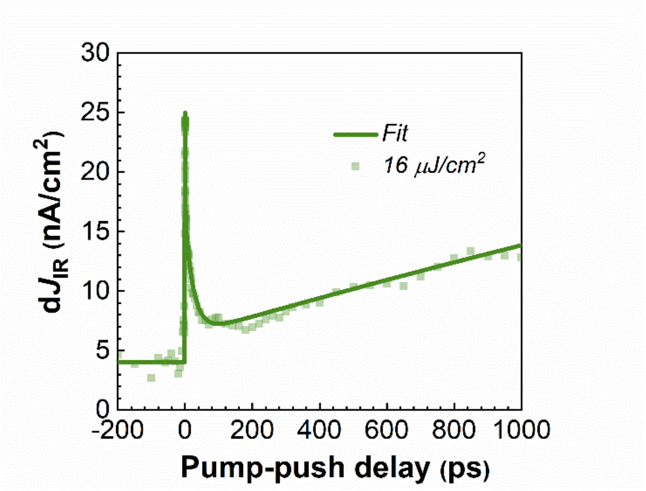

Fig. S4 Fs PPPC carrier dynamics of $BiVO_4$ under short-circuit conditions. The $BiVO_4$ films was illuminated under 450 nm pump (16 µJ/cm^2) and 1200 nm push (0.3 mJ/cm^2) from the backside of sample in 0.1 M phosphate buffer (pH 7).

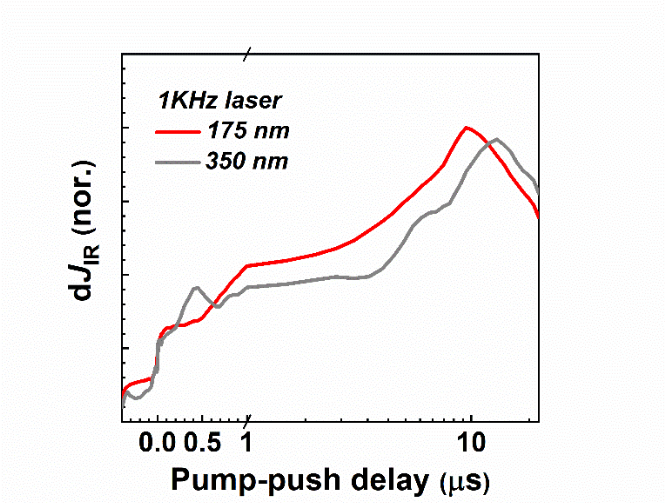

Fig.S5 Push-induced PPPC carrier dynamics of 350 nm and 175 nm $BiVO_4$ under short-circuit condition. The $BiVO_4$ films was excited with a Vis 400 nm pump and an IR 1064 nm push from the backside of sample in 0.1 M phosphate buffer (pH 7).

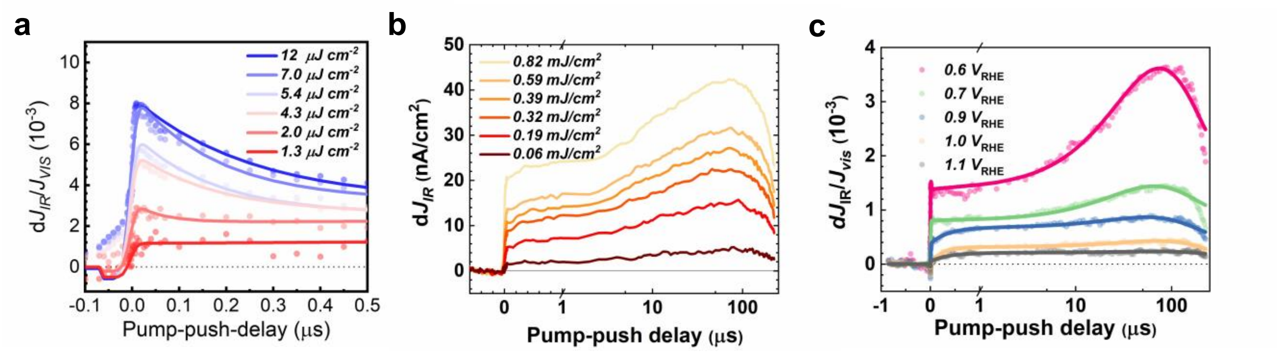

Fig. S6 (a) Pump intensity-dependence of early time pump-push-photocurrent dynamics in Figure 4a. **(b)** Push intensity-dependence of the pump-push-photocurrent dynamics. The sample was excited with a Vis 405 nm (1.3 μJ/cm^2) pump and an IR 1064 nm push of varying intensities from the back side under under 0.6V$_{RHE}$ **(c)** Bias-dependent pump-push-photocurrent dynamics normalized by the pump-induced photocurrent. The film was excited with a 405 nm (1.3μJ cm^{-2}) pump and a 1064 nm (0.5 mJ cm^{-2}) push. All experiments are measured with the $BiVO_4$ photoelectrode in a two-electrode configuration in 0.1 M phosphate buffer (pH 7).

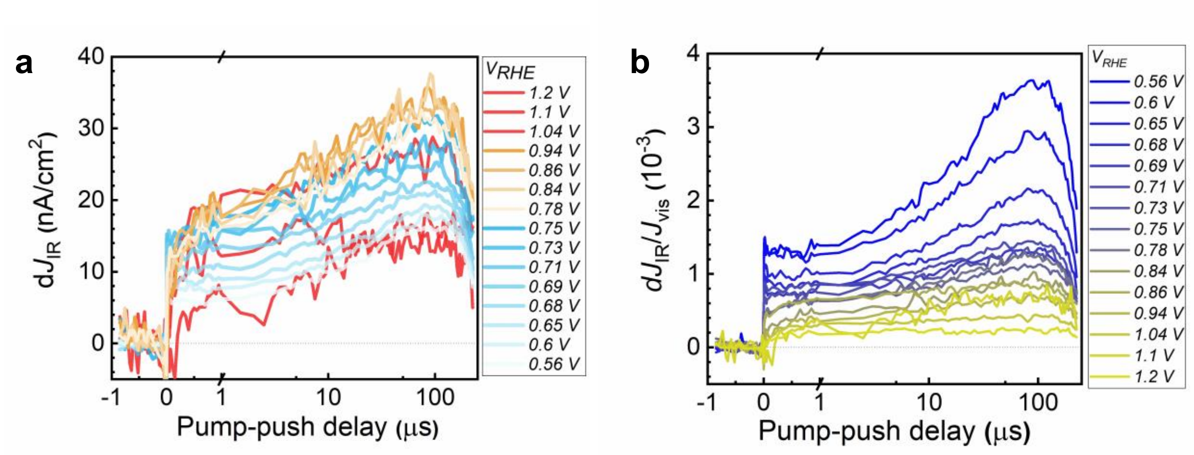

Fig. S7 (a) Bias-dependent pump-push-photocurrent dynamics under working conditions from 0.56 V to 1.2 V$_{RHE}$. **(b)** The dynamics normalized by the pump-induced photocurrent. The film was excited with a 405 nm (1.3 μJ cm^{-2}) pump and a 1064 nm (0.3 mJ cm^{-2}) push. All experiments are measured with the $BiVO_4$ photoelectrode in a two-electrode configuration under short-circuit conditions in 0.1 M phosphate buffer (pH 7) with back illumination.

2. Fitting of the PPPC data:

We analyze the continuous wave PPPC (Fig. S3(a)) recombination process by a Cole-Cole function (equation 1), which are normally used to describe complex dispersive exponential decays in amorphous solids with high densities of electronic traps.

$$R(w) = \frac{R_0}{1 + (iw\tau_0)^{\alpha}} \qquad \text{EqS1}$$

where $\alpha<1$ and indicates the dispersive extent of the decay, τ_0 is the carrier lifetime and R_0 is the response under steady state with zero chopping freq. R is written as a complex function, the in phase and out phase component are real and imaginary part respectively.

The Transient PPPC dynamics in this work under different conditions are fitted by a function of convoluted gaussian and exponential decay/growth:

$$F(x) = \int_{-\infty}^{+\infty} \left\{ \frac{e^{-\frac{t^2}{2\sigma^2}}}{\sigma\sqrt{2\pi}} \times \left[e^{-\frac{(x-x_0)-t}{Dec\,\tau_i}} \times \left(1 - e^{-\frac{(x-x_0)-t}{Ing\,\tau_i}}\right)\right]\right\} \partial t \qquad \text{EqS2}$$

Where X_0 is time zero, σ is Gauss Sigma (decided by laser time resolution), Decay Exp Tau = $Dec\,\tau_i$, Ingrow Exp Tau = $Ing\,\tau_i$. Multiple decay processes may appear with different conditions. Derived lifetime and errors shown below.

Table. S1 Carrier growth and decay lifetime of different pump intensity (Fig. 4a). All experiments are measured in a home designed PEC cell.

Intensity (μJ/cm^2)	Decay $\tau0$ (μs)	Rise $\tau1$ (μs)	Decay $\tau2$ (μs)
12.00	0.25 ± 0.03	121.80 ± 27.51	190.80 ± 90.77
7.00	0.18 ± 0.02	100.70± 18.21	141.20 ± 41.15
5.40	0.17 ± 0.03	88.28 ± 1.00	111.60 ± 24.84
4.30	0.17 ± 0.03	63.61 ± 6.56	74.89 ± 8.90
2.00	0.06 ± 0.03	55.23 ± 5.09	62.88 ± 6.51
1.30	NA*	61.50 ± 9.81	145.30 ± 1.40

*There is no decay component at early time under low intensity

Table. S2 Carrier growth and decay lifetime of different bias (Fig. 4b). All experiments are measured in a home designed PEC cell.

External bias (vs Pt)	Fast Rise $\tau 3$ (μs)	Slow Rise $\tau 4$ (μs)	Decay $\tau 5$ (μs)
0.00	<0.01	61.50 ± 9.81	145.30 ± 1.40
0.20	<0.01	43.47 ± 1.28	153.10 ± 3.18
0.60	0.35 ± 0.05	66.34 ± 0.30	144.90 ± 0.04
1.00	0.18 ± 0.01	71.99 ± 1.59	275.90 ± 1.01
1.20	0.36 ± 0.05	111.86 ± 2.69	311.72 ±3.84
